# The silent warning: High-sensitivity cardiac troponin as a biomarker for subclinical coronary artery disease in seemingly healthy Japanese population

**DOI:** 10.1016/j.pmedr.2026.103516

**Published:** 2026-06-01

**Authors:** Ryohei Suganuma, Toshio Shimada, Akihiro Sonoda, Daiki Murakoshi, Naoki Hiramatsu, Toru Sabashi, Shin Koga

**Affiliations:** a Department of Clinical Laboratory Medicine, Shizuoka General Hospital, Kita-Ando 4-27-1, Aoi-Ku, Shizuoka City, Shizuoka, Japan; b Research Support Center, Shizuoka General Hospital, Kita-Ando 4-27-1, Aoi-Ku, Shizuoka City, Shizuoka, Japan; c Shizuoka Health Promotion Center, Shizuoka Broadcasting System, Toro 3-1-1, Suruga-ku, Shizuoka City, Shizuoka, Japan

**Keywords:** Cardiac troponin, Sex difference, Coronary heart disease, Suita score, Framingham score

## Abstract

**Objectives:**

High-sensitivity cardiac troponin (cTn) assays are established for diagnosing acute myocardial infarction, but their distribution and clinical relevance in healthy Japanese populations remain unclear. This study clarified sex differences in high-sensitivity cTnI and cTnT levels and evaluated their associations with estimated 10-year coronary heart disease (CHD) risk in a healthy Japanese population.

**Methods:**

We conducted the study at the Shizuoka Broadcasting Service Shizuoka Health Promotion Center between December 2012 and March 2013 and included 808 participants (541 males and 267 females) with no history of CHD. Estimated 10-year CHD risks were calculated using the Framingham and Suita risk scores. Multivariable linear regression analyses were performed using log-transformed cTn levels as dependent variables and cardiovascular risk factors as explanatory variables. We compared the estimated 10-year incidence of CHD across groups, divided by troponin quartile.

**Results:**

Both cTnI and cTnT levels were significantly higher in males than in females. No significant multicollinearity was observed (all variance inflation factors <2.0). Group comparisons showed that the estimated 10-year CHD risk increased stepwise with higher cTn quartiles.

**Conclusions:**

High-sensitivity cTnI and cTnT levels are independently associated with estimated 10-year CHD risk scores in a healthy Japanese population.

## 1 Introduction

We have widely used high-sensitivity cardiac troponin T (cTnT) and I (cTnI) to diagnose acute myocardial infarction (AMI). These assays have enabled us to diagnose AMI more quickly and accurately (Thygesen et al., 2012; Reichlin et al., 2009; Weber et al., 2011).

Furthermore, many studies have examined the use of serum cardiac troponin (cTn) to diagnose AMI, but few have examined it in healthy populations. Although sex-specific differences in serum cTn levels in healthy populations have been examined by many studies (Apple et al., 2012; Collinson et al., 2012; Koerbin et al., 2013), few studies have examined these differences in Japanese populations. Therefore, this study aimed to clarify the characteristics of cTn levels and sex differences in cTn levels in a healthy Japanese population.

## 2 Methods

### 2.1 Study design and population

This study is a cross-sectional study with periodic health examination data.

We implemented the study using non-connectable, anonymous data from 818 participants (549 males and 269 females) who underwent a periodic health examination at the Shizuoka Broadcasting Service Shizuoka Health Promotion Center in December 2012 to March 2013. We excluded 10 participants due to a history of coronary heart disease (CHD). The characteristics of 808 participants (541 males and 267 females) are shown in Table 1. The flow diagram for sample size is shown in Supplementary Fig. 1.

**Table 1:** Characteristics of participants and results of a multiple regression analysis using factors used to calculate the Framingham and Suita scores as explanatory variables and cardiac troponin I and T levels as the response variable for 808 adults, the Shizuoka Broadcasting Service Shizuoka Health Promotion Center, December 2012 to March 2013.

	Total *n* = 808	Male *n* = 541	Female *n* = 267	Cardiac troponin I		Cardiac troponin T		VIF
Factor				Standardized coefficient	*p*-value	Standardized coefficient	*p*-value	
Age, years	48.4 ± 8.2	49.0 ± 8.3	47.1 ± 7.7	0.22	<0.01	0.29	<0.01	1.12
Current smoker, n (%)	192 (23.8)	176 (21.8)	16 (2.0)	0.01	0.66	0.05	0.07	1.11
Diabetes, n (%)	49 (6.1)	39 (4.8)	10 (1.2)	0.03	0.36	0.10	<0.01	1.04
Dyslipidemia, n (%)	340 (42.1)	264 (32.7)	76 (9.4)	0.05	0.13	−0.01	0.74	1.05
Chronic kidney disease, n (%)	45 (5.6)	36 (4.5)	9 (1.1)	0.01	0.72	0.08	<0.01	1.05
Sex (Male), n (%)	541 (67.0)	541 (100.0)	0 (0.0)	0.32	<0.01	0.46	<0.01	1.16
Hypertension, n (%)	60 (7.4)	55 (6.8)	5 (0.6)	0.10	<0.01	0.07	0.02	1.04

Abbreviations: VIF, variance inflation factor. Values are expressed as mean ± standard deviation or number (percentage). The p-value is for the standardized coefficient of each explanatory variable.

In this study, we determined whether the participant had hypertension, dyslipidemia, diabetes, and chronic kidney disease (CKD) using periodic health examination data or subject-reported history. We adopted the diagnostic criteria for hypertension from the Japanese Society of Hypertension and dyslipidemia from the Japan Atherosclerosis Society. Diabetes was defined according to the Japan Diabetes Society criteria for “diabetic type” (HbA1c ≥ 6.5% or fasting blood glucose ≥126 mg/dL) or a history of diabetes. The criteria for “diabetic type” are in agreement with the American Diabetes Association's diagnostic criteria for diabetes. We applied the Japan Society of Nephrology's criteria for CKD. This criterion acknowledges that we use the estimated glomerular filtration rate (eGFR) from serum creatinine in routine clinical practice. Therefore, we used eGFR to determine whether participants have CKD.

First, we investigated the strength of the association between CHD risk factors and cTn levels in Japanese individuals without a history of CHD.

Second, we calculated the 99th percentile for cTn in each sex among the 318 healthy participants (156 males and 162 females). We defined the “healthy individual” as meeting the following inclusion criteria: no history of cardiovascular or cerebrovascular disease, an eGFR of 60 mL/min/1.73 m^2^ or higher, absence of hypertension, dyslipidemia, or diabetes, and an almost normal electrocardiogram pattern. These inclusion criteria were based on the IFCC Task Force on Clinical Applications of Cardiac Bio-Markers paper (Apple et al., 2017).

Third, we examined the correlation between the estimated probability of CHD incidence from risk scores and serum cTn levels in 808 participants (541 males and 267 females) without a history of CHD. We divided participants into quartiles by serum cTn levels, from Q1 (the lowest: 25th percentile or below) to Q4 (the highest: above the 75th percentile, up to the 99th percentile). These quartiles are based on the quartiles obtained from 808 participants without a history of CHD (the “All” row in Supplementary Table 1). We used the Framingham risk score (Wilson et al., 1998) and the Suita risk score (Nishimura et al., 2014) to estimate each participant's 10-year risk of CHD. We calculated both risk scores using low-density lipoprotein cholesterol (LDL-C) instead of total cholesterol. We calculated LDL-C levels using Friedewald's equation. However, 10 males with triglyceride concentrations above 400 mg/dL used a direct LDL-C assay.

This study was conducted in accordance with the Declaration of Helsinki and was approved by the Shizuoka General Hospital Research Ethical Committee approved this study (SGHIRB#2016056). All data handling and privacy protections were conducted in strict compliance with the guidelines of the institution.

### 2.2 Measures

We measured high-sensitivity cTnI using the Architect^R^ i2000 Analyzer (Abbott Diagnostics, Abbott Park, IL, USA). The analytical performance is as follows: limit of detection (LoD) is 1.1 ng/L, Concentration at 20% coefficient of variation (limit of quantification, LoQ) is 1.3 ng/L, and coefficient of variation (%CV) at the overall 99th percentile is 4.0% (Collinson et al., 2019).

We used it to measure high-sensitivity cTnT using a Modular Analytics E170 Analyzer (Roche Diagnostics, Mannheim, Germany). The analytical performances are as follows: LoD is 2.05 ng/L, LoQ is 2.20 ng/L, and the %CV at the overall 99th percentile is below 10% (Collinson et al., 2019). For participants with cTnT concentrations below 3.0 ng/L, cTnT levels were estimated from the calibration curve and signal values. Among participants with serum high-sensitivity cTnT below 3.0 ng/L, 11 were male, and 99 were female.

### 2.3 Statistical analysis

We performed a multiple linear regression analysis to determine the strength of the association between each factor and serum cTn levels in these participants. The explanatory variables were based on the factors used to calculate the Framingham and Suita scores (age, current smoking status, diabetes, dyslipidemia, CKD, hypertension, and sex (Wilson et al., 1998; Nishimura et al., 2014)), and the objective variables are log-transformed cTnI or cTnT.

We compared the estimated probabilities of 10-year CHD incidence using the Framingham and Suita risk scores across four groups (Q1-Q4) using the Kruskal-Wallis test. We performed a group comparison using the Steel-Dwass test. Additionally, we used the Jonckheere-Terpstra test to assess whether higher cTn levels were associated with higher 10-year CHD incidence rates estimated using the Framingham or Suita risk scores.

We performed all statistical analyses using R (The R Foundation for Statistical Computing, Vienna, Austria, version 4.5.0) and its graphical user interface EZR (Saitama Medical Center, Jichi Medical University, Saitama, Japan, version 1.55) (Kanda, 2013). More precisely, EZR is a modified version of R Commander (version 2.9–5) that adds statistical functions frequently used in biostatistics.

## 3 Results

Table 1 shows the strength of association between CHD risk factors and cTn levels. The standardized coefficient of sex was 0.325 (*p* < 0.01) on the log cTnI level and 0.458 (*p* < 0.01) on the log cTnT level. Age and hypertension are significantly associated with both cTnI and cTnT levels. Diabetes and CKD are significantly associated with only cTnT. Multicollinearity among the explanatory variables was assessed using the variance inflation factor (VIF), and all VIF values were confirmed to be less than 2.0, indicating no significant multicollinearity in the regression model.

Supplementary Table 1 presents the 99th percentile values for the healthy population, stratified by sex. The 99th percentile values for cTnI calculated from the healthy population were 21.1 ng/L in males and 9.0 ng/L in females. The 99th percentile values for cTnT were 11 ng/L for males and 9 ng/L for females.

**Fig. 1:**
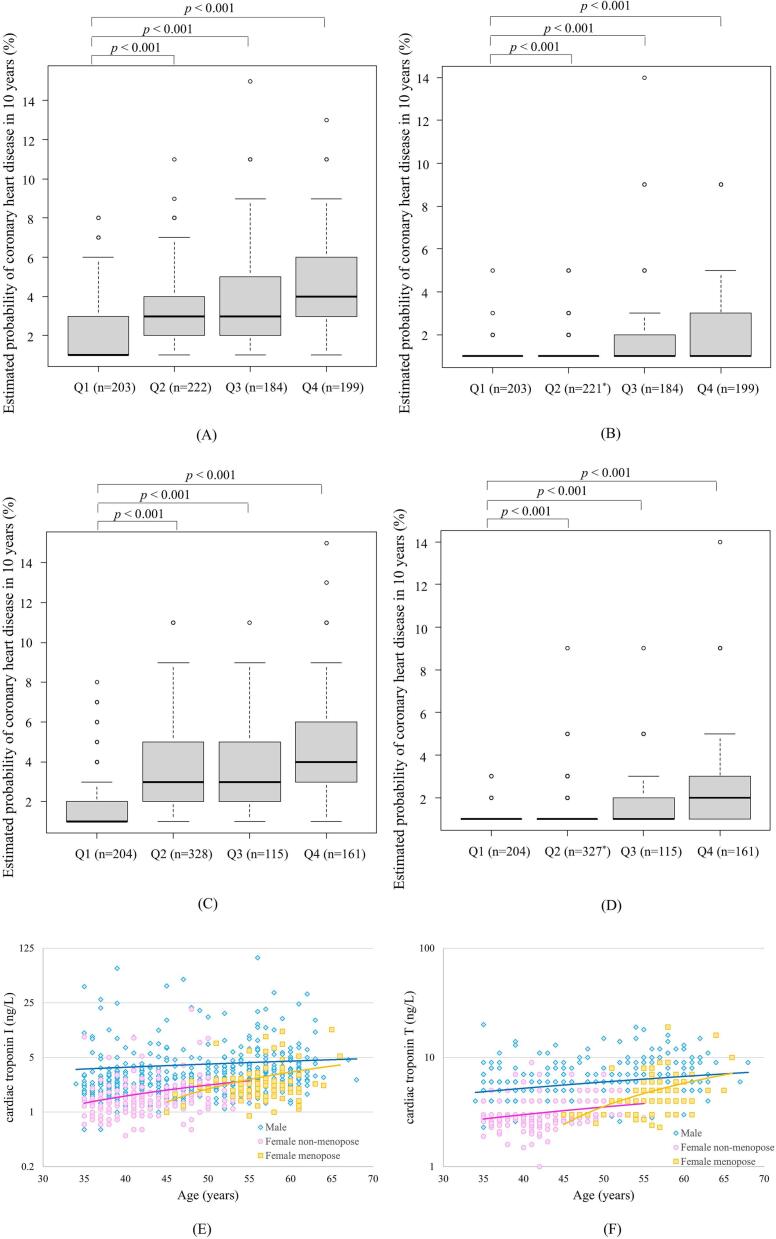
Stratification of cardiac troponin levels and their associations with estimated coronary heart disease risk scores and age in 808 adults, the Shizuoka Broadcasting Service Shizuoka Health Promotion Center, December 2012 to March 2013. (A–D) Comparison of estimated 10-year coronary heart disease risk scores across quartiles of high-sensitivity cardiac troponin I and cardiac troponin T levels. Coronary heart disease risk was estimated using the Framingham and Suita risk scores. We used the Kruskal-Wallis and the Steel-Dwass test for statistical analysis: (A) Framingham score vs. cardiac troponin I, (B) Suita score vs. cardiac troponin I, (C) Framingham score vs. cardiac troponin T, and (D) Suita score vs. cardiac troponin T, (*E*-F) Scatter plot showing the correlation between age and log-transformed cardiac troponin levels. The solid lines represent the regression lines between age and troponin concentration, separated by sex and pre-menopausal/post-menopausal group: (E) cardiac troponin I and age, (F) cardiac troponin T and age. * One subject was excluded because she was too young to calculate a Suita score.

Fig. 1(A)–(D) shows the comparison of the estimated 10-year CHD incidence probability across four groups, divided by quartiles of serum cTnI levels. The Q3 and Q4 groups have a significantly higher likelihood of CHD incidence than the Q1 group. Similar results were obtained when participants were divided into four groups based on serum cTnT levels. Furthermore, the Jonckheere-Terpstra test confirmed that the estimated probability of CHD incidence increased monotonically with cTnI levels for both the Framingham score (*p* < 0.01) and the Suita score (*p* < 0.01). Similarly, the estimated probability of CHD incidence increased monotonically with cTnT levels for both the Framingham score (*p* < 0.01) and the Suita score (*p* < 0.01).

## 4 Discussion

The concept that an elevation of serum cTn level in a patient indicates a poor or ominous prognosis has already been accepted in clinical practice, regardless of the reasons (Daniels et al., 2008; Xue et al., 2011). However, our study suggests that the difference in cTn levels among healthy individuals may also have clinical significance.

In our study, quartile stratification by serum cTn levels among participants showed that Q3 and Q4 (the upper two groups) had significantly higher CHD risk scores than Q1. These results suggest that serum cTn levels may correlate with one's probability of CHD incidence in the population without a history of CHD.

Sex is a significant factor in both cTn levels and CHD incidence. Our study, as in other populations, shows that serum cTnI and cTnT levels were significantly higher in males than in females in the Japanese population.

From an anatomical perspective, this difference may be due to variations in myocardial mass (Koerbin et al., 2013; Shah et al., 2015). The left ventricle is significantly larger in males than in females (de Simone et al., 1995; de la Grandmaison et al., 2001). So, sex differences may result from this difference. In recent years, a similar study of Japanese populations reported that male hearts weigh more than female hearts in absolute heart weight, heart weight per body weight, and heart weight per height (Kakimoto et al., 2021). Since males have more myocardial muscle that releases troponin than females do, it can be inferred that their troponin levels are higher.

From another perspective, biological differences between males and females may contribute. Fig. 1 (E) and (F) shows a correlation between age and serum cTnI or cTnT levels. This figure shows that males have higher serum cTn levels than females. However, the slope of the regression line is steeper in females than in males, and the sex difference in serum cTn levels diminishes with age. Furthermore, the slope was steeper in postmenopausal females than in premenopausal females. Due to the limited data in this study, it is not possible to draw definitive conclusions; however, we believe the relationship between cTn and sex differences warrants further investigation.

The most clinically relevant question is why cTn levels are associated with other risk factors. In this study, sex is the most influential factor, but cTn levels are also associated with the other factors.

The previous research has shown that the high-sensitivity cTnI test can predict carotid atherosclerosis at a subclinical stage (Lyngbakken et al., 2021). Similarly, it can be inferred that cTn levels may reflect plaque progression. A similar argument has been made in a previous study (Apple et al., 2003), but this idea remains speculative because coronary plaque proliferation was not directly measured in our study.

We hope that these questions will be answered by larger-scale prospective studies.

### 4.1 The limitations of this study and the challenges expected in the future

This cross-sectional study lacks time-course data, which significantly limits the evidence. In this study, the primary outcome is defined not as the actual incidence rate of CHD but as the probability of developing CHD, calculated using a risk score. Further prospective studies are needed to investigate the association between cTn levels and the actual risk of developing CHD. Furthermore, to confirm the clinical significance of elevated cTn levels in healthy individuals, a larger sample size, particularly among high-risk groups, is required.

The number of healthy participants is also a limitation. The IFCC guideline requires 300 males and 300 females to determine 99th percentile values (Apple et al., 2017). However, our study includes fewer than 300 healthy individuals of each sex. Larger-scale studies are needed to calculate the 99th percentile value of the accurate cTn value in the Japanese population.

## 5 Conclusions

cTn levels may be associated with the risk of CHD incidence in individuals with no history of CHD.

Since serum cTn levels are significantly influenced by sex, the patient's sex should be considered when diagnosing CHD in the Japanese population as well.

## CRediT authorship contribution statement

**Ryohei Suganuma:** Writing – original draft, Methodology, Formal analysis, Data curation, Conceptualization, Investigation, Visualization. **Toshio Shimada:** Writing – review & editing, Project administration, Methodology. **Akihiro Sonoda:** Writing – review & editing. **Daiki Murakoshi:** Resources, Data curation, Writing – review & editing. **Naoki Hiramatsu:** Resources, Writing – review & editing. **Toru Sabashi:** Supervision, Investigation, Writing – review & editing. **Shin Koga:** Supervision, Investigation, Writing – review & editing.

## Declaration of competing interest

The authors declare that they have no known competing financial interests or personal relationships that could have appeared to influence the work reported in this paper.

## Data Availability

Data will be made available on request.
