# Supplementary material for: The silent warning: High-sensitivity cardiac troponin as a biomarker for subclinical coronary artery disease in seemingly healthy Japanese population
Source: Prev Med Rep. 2026 Jun 1;67:103516. doi: 10.1016/j.pmedr.2026.103516 (PMC13267694; doi:10.1016/j.pmedr.2026.103516)
Supplement: Supplementary file 1 — Supplementary material [file mmc1.docx]

Patients who underwent a periodic health examination at the Shizuoka Broadcasting Service Shizuoka Health Promotion Center in December 2012 to March 2013

(n=818; 549 males and 269 females)

Excluded

Having coronary heart disease history

(n=10; 8 males and 2 females)

Without a CHD history

(n=808; 541 males and 267 females)

Healthy population

(n=318; 156 males and 162 females)

Excluded

Not meeting “healthy individuals” criteria in this study

(n=490; 385 males and 105 females)

Supplementary Figure 1. Flow diagram of participant selection for the study at the Shizuoka Broadcasting Service Shizuoka Health Promotion Center, December 2012 to March 2013.

Supplementary Table 1. Quartile value of cardiac troponin calculated from 808 participants who underwent a periodic health examination at the Shizuoka Broadcasting Service Shizuoka Health Promotion Center, December 2012 to March 2013.

|  |  | 99^th^  percentile | 75^th^  percentile | Median | 25^th^  percentile |
| --- | --- | --- | --- | --- | --- |
| All  (N=808) | Cardiac troponin T (ng/L) | 15 | 6 | 5 | 3 |
|  | Cardiac troponin I (ng/L) | 24.7 | 3.2 | 2.3 | 1.6 |
| Male  (N=541) | Cardiac troponin T (ng/L) | 11 | 6 | 5 | 4 |
|  | Cardiac troponin I (ng/L) | 21.1 | 3.3 | 2.4 | 1.7 |
| Female  (N=267) | Cardiac troponin T (ng/L) | 9 | 4 | 3 | 2.6 |
|  | Cardiac troponin I (ng/L) | 9.0 | 1.9 | 1.4 | 1.0 |
